# Carotenoid-Enriched Fractions From *Spondias mombin* Demonstrate HER2 ATP Kinase Domain Inhibition: Computational and *In Vivo* Animal Model of Breast Carcinoma Studies

**DOI:** 10.3389/fonc.2021.687190

**Published:** 2021-08-31

**Authors:** Damilohun Samuel Metibemu, Oluseyi Adeboye Akinloye, Idowu Olaposi Omotuyi, Jude Ogechukwu Okoye, Mustapha Ayodele Popoola, Adio Jamiu Akamo

**Affiliations:** ^1^Department of Biochemistry, Adekunle Ajasin University, Akungba-Akoko, Nigeria; ^2^Department of Biochemistry, Federal University of Agriculture, Abeokuta, Nigeria; ^3^Institute for Drug Research and Development, Afe Babalola University, Ado Ekiti, Nigeria; ^4^Department of Medical Laboratory Science, Faculty of Health Sciences and Technology, College of Health Sciences, Nnamdi Azikiwe University, Nnewi, Nigeria; ^5^Research and Development Desk, Office of the Executive Secretary Tertiary Education Trust Fund, Abuja, Nigeria

**Keywords:** human epidermal growth factor 2, liquid chromatography/mass spectrometry, 7,12-dimethylbenz[*a*]anthracene (DMBA), breast cancer, carotenoids

## Abstract

Human epidermal growth factor 2 (HER2) is overexpressed in about 20% of breast cancer and is associated with a poor prognosis. We report in this study that carotenoid-enriched fractions from *Spondias mombin* demonstrate HER2 ATP kinase domain inhibition. HER2 breast carcinoma was modeled in female Wistar rats and authenticated *via* immunohistochemical studies. Inhibition of HER2 ATP kinase domain by the carotenoid-enriched fractions was investigated by molecular docking, atomistic simulation, and the expression of HER2 mRNA in HER2-positive breast carcinoma model in female Wistar rats. The therapeutic efficacy of the treatments (carotenoid-rich fractions) was determined by biochemical, tumor volume, and histopathological analysis. Immunohistochemical analysis revealed 7,12-dimethylbenz[*a*]anthracene (DMBA)-induced HER2-positive breast carcinoma. Phytoconstituents of the carotenoid-enriched fractions astaxanthin, 7,7′,8,8′-tetrahydro-β,β-carotene, beta-carotene-15,15′-epoxide, and lapatinib (standard drug) demonstrate inhibition of HER2 with docking scores of −3.0, −8.5, −11.5, and −10.6 kcal/mol, respectively; and during atomistic simulation, the compounds ruptured the canonical active-state K753/E770 salt-bridge interaction. The treatment similarly downregulated HER2 mRNA expression significantly at *p* < 0.05. It also upregulated the expression of p53 and p27 mRNAs significantly at *p* < 0.05 and reduced creatinine and urea concentrations in the serum at *p* < 0.05. The tumor volume was also significantly reduced when compared with that of the untreated group. Carotenoid-enriched fractions from *S. mombin* demonstrate anti-HER2 positive breast carcinoma potentials *via* HER2 ATP kinase domain inhibition.

## Introduction

Breast cancer represents the most common form of cancer in women and the second most diagnosed cancer after lung cancer (11.6% of all cancer cases). An average of not less than 42,000 women dies of breast cancer every year in the United States (1). It is undoubtedly the second-leading cause of cancer-related mortality in women (1). According to DeSantis et al. (2), the propensity of dying from breast cancer in a lifetime is approximately 2.6%. There are three molecular classes of breast cancer: hormone receptor positive/ERBB2 (receptor tyrosine-protein kinase)-negative (HR^+^/ERBB2^−^), ERBB2 positive (ERBB2^+^), and triple-negative (3). Epidermal growth factor 2 (ERBB2), formerly known as HER2 or HER2/neu), is a receptor tyrosine kinase that belongs to the epidermal growth factor receptor family. It is overexpressed in about 20% of breast cancer and is associated with a poor prognosis (4). Breast tumors with the overexpression of ERBB2 genes are said to be ERBB2^+^ (HER2^+^) (5). Knowledge of the types of breast cancer helps in determining the best forms of treatments and prognosis. Patients with the overexpression of ERBB2 can be treated with ERBB2-targeted therapy: monoclonal antibody (mAbs) drugs, trastuzumab, pertuzumab, and small-molecule inhibitors, lapatinib and neratinib (5). Treatment of HER2^+^ breast cancer with chemotherapy and radiotherapy is associated with different complications and unpleasant side effects (6). Plant-derived phytochemicals are known to demonstrate excellent anti-neoplastic properties (7, 8). These include the carotenoids; the dietary intake of carotenoids corresponds to reduced cancer risk (9). Reports reveal that carotenoids could hinder the growth of neoplastic cells. They have therapeutic benefits in some cancers (10, 11).

We report in this study that carotenoid-enriched fractions from *Spondias mombin* leaves demonstrate anti-HER2 positive breast carcinoma potentials *via* HER2 ATP kinase domain inhibition.

## Materials and Methods

### The Plant Materials

The leaves of *S. mombin* were from the Federal University of Agriculture, Abeokuta campus, and stored at room temperature (with limited oxygen and in the dark) for 4 months to allow the leaves to dry. They were certified at the Department of Applied Botany, Federal University of Agriculture, Abeokuta, Nigeria, and pulverized into powder.

### Extraction and Isolation of Carotenoids

Carotenoids were extracted and isolated following the methods of Rodriguez (12) and Metibemu et al. (8), respectively. *N*-Hexane/acetone [1:1 (v/v)] (2,500 L) was used to soak the pulverized leaves (500 g) for 24 h and filtered with Whatman no. 42 filter paper. KOH (potassium hydroxide) of 40% (w/v) in methanol was added to the filtrate. Salting out was carried out by addition of 10% (w/v) Na_2_SO_4_ (sodium sulfate). The solution was introduced into a separating funnel. The bottom layer was removed after it had separated into two layers. The upper layer was washed three times. Anhydrous Na_2_SO_4_ (powder form) was added, and the solution was filtered. The filtrate was saved. Silica gel (230–240 mesh size) prepared with 4% (v/v) acetone in hexane was used to fill a column, while the top of the silica gel was filled with 2-cm layer of anhydrous Na_2_SO_4_. The filtrate from above was poured into the column and eluted with 4% (v/v) acetone in hexane in the first instance. Acetone/hexane 1:9 (v/v) and 1:8 (v/v) acetone/hexane were also used to elute, respectively.

### Liquid Chromatography/Mass Spectrometry and Electrospray Ionization

An ultra-high-performance liquid chromatography–quadrupole time-of-flight–mass spectrometry (UHPLC–QTOF–MS) method was set up for the screening, with typical injection volumes of 50 μl of extract. Separation was performed on a Dionex Ultimate 3000 UHPLC system (Thermo Scientific, Dionex, Sunnyvale, CA, USA) equipped with a RaptorTM ARC-18 2.7 µm 100 × 2.1 mm column, held at a temperature of 25°C, and using a gradient system composed of A: 0.1% formic acid in water, and B: 0.1% formic acid in methanol. The flow was maintained at 0.3 ml min^−1^ throughout the run. The developed gradient program was 70% to 50% A in 3 min (hold 2 min), 50% to 20% A in 2 min (hold for 2 min), and 20% to 95% A in 2 min (hold for 3 min).

TOF detection was performed using a compact QTOF orthogonal mass spectrometer (Bruker Daltonics, Bremen, Germany) operated at a resolving power of ~23,000 full width at half maximum (FWHM). The instrument was equipped with an orthogonal electrospray ionization (ESI) source, operated at positive mode, and autoMSMS spectra were recorded in the range *m*/*z* 50–3000 with the collision energy of 20–50 eV, with five scans per second. For calibration, 1 μl 10 mmol L^−1^ sodium formate was injected at the beginning of each chromatographic run, using the divert valve (0.3–0.4 min).

ESI+ capillary voltage was maintained at 5,000 V, the gas flow to the nebulizer was set to 1.8 bar, the drying temperature was 220°C, and the drying gas flow was 9.0 L min^−1^.

### Docking of Carotenoid-Enriched Fractions Against HER2 and Molecular Dynamics Simulation Studies

Phytoconstituents of the carotenoid-enriched fractions (8) were retrieved from the PubChem repository in the structure-data format (SDF) and changed to PDB. They were docked against the kinase domain of human HER2 (erbB2) (3PP0) using Autodock 4.0 suites. Water molecules within 3PP0 ATP kinase domain were deleted; and the grid coordinates (X = 17.1, Y = 16.55, Z = 26.6) of the co-crystallized, 2-{2-[4-({5-chloro-6-[3-(trifluoromethyl)phenoxy]pyridin-3-yl}amino)-5*H*-pyrrolo[3,2-*d*]pyrimidin-5-yl]ethoxy}ethanol were adopted for docking. The co-crystallized ligand was re-docked within the 3PP0 ATP kinase domain to validate the docking scores. In order to further validate that the compounds selected following docking were inhibitors of human HER2, an active state 3D structure was generated using the template (PDB ID: 5JEB, this structure has fully formed salt bridge between K753/E770 (NZ—OE_2_ distance = 2.6 Å (13). Four starting biosystems were prepared (APO state, lapatinib-bound (LAP), beta-carotene-15,15'-epoxide-bond (BTC), and 7′78′8′-tetrahydrobeta-beta-carotene-bound (TTC) complexes) using CHARMM-GUI (14). All protein atoms were parameterized using CHARMM36 all-atom additive protein force field (15) while the ligands were parameterized using (https://cgenff.paramchem.org/) as implemented on CHARMM-GUI. Each complex was fully immersed in an octahedral box of TIP3P water model. All simulations were performed on HPC clusters at the Institute for Drug Research and Development, Afe Babalola University. All biosystems were simulated using periodic boundary conditions, and long-range electrostatic interactions were estimated using Ewald summation (16), while the SHAKE algorithm was used to treat the bonds involving hydrogen atoms (17). The temperature (Berendsen thermostat) and pressure (barostat) of the systems were maintained, while integration of Newton’s equations (2-fs time step) during equilibration (5 ns, NVT ensemble, and 15-ns NPT ensemble) and production (50 ns) molecular dynamics (MD) simulations as previously reported (18). Sampled conformations along the trajectories were collected every 50 ps for analysis.

Root-mean-square deviation (RMSD) from the starting structure was used to confirm convergence (data not shown), while inter-atomic distance between K753 (Cα) and E770 (Cα) was calculated using PLUMED-plugin in VMD (19) and plotted as frequency distribution (GraphPad Prism ver. 9.0). The binding energy was calculated using molecular mechanics/generalized Born surface area (MM/GBSA) method as implemented in MOLALCAL suite (19).

### HER2^+^ Breast Carcinoma Model and Experimental Design

HER2^+^ breast carcinoma model was by a single intraperitoneal administration of 7,12-dimethylbenz[*a*]anthracene (DMBA) (35 mg/kg body weight). The tumor volume was by Geran et al. (20) method. The female Wistar rats (21), 50 to 60 days old, were acclimatized for 2 weeks. The protocols and the procedures of the experiment were endorsed by the Federal University of Agriculture, Abeokuta, Animal Ethics Board, with approval FUNAAB/IAEB/1911108. The Wistar rats were separated into six groups of five rats per group.

Group 1 received standard chow. Group 2 received 35 mg/kg body weight of DMBA intraperitoneally. Groups 3 and 4 received 35 mg/kg body weight of DMBA and treated with 100 and 200 mg/kg carotenoid-enriched fractions, p.o. for 28 days, after tumor formation.

Group 5 Wistar rats received 35 mg/kg body weight of DMBA. After tumor formation, they were given carotenoid-enriched fractions (100 mg/kg) and 100 mg/kg celecoxib p.o. for 28 days. Group 6 Wistar rats received 200 mg/kg body weight of carotenoid-enriched fractions only, p.o. for 28 days.

Note:

- Per os (p.o.): administration *via* gavage.
- Treatment with carotenoid-enriched fractions commenced simultaneously in group 6 and groups 3–5.
- Tumor formation was in all the groups (3–5) before treatment with carotenoid-enriched fractions.
- COX-2 expression corresponds to angiogenesis (22). In group 5, celecoxib was adopted to access the dual inhibition of the HER2 kinase domain and COX-2.
- The mammary tumor was formed in the Wistar rats after 24 weeks following DMBA administration.

$$\text{Tumour Volume}=\frac{\text{length }(\text{cm})\times \text{widt}{\text{h}}^{2}(\text{cm})}{2}$$

### Immunohistochemistry Validation of HER2^+^ Breast Carcinoma

The avidin–biotin complex (ABC) method was employed. The antibody dilution factor of 1:100 dilution was used for all the antibody markers. The processed breast tumors were sectioned at 2 µm on the rotary microtome and placed on the hot plate at 70°C for 1 h. The sectioned tissues were immersed in water through three changes of descending grades of alcohol and finally to water. Antigen retrieval was performed by heating them on a citric acid solution of pH 6.0 using a microwave at power 100 for 15 min. The sectioned tissues were equilibrated gradually with cool water to displace hot citric acid for at least 5 min for cooling. Peroxidase blocking was performed by covering the tissues with 3% hydrogen peroxide (H_2_O_2_) for 15 min and cleaned with phosphate-buffered saline (PBS). Protein blocking by avidin lasted for 15 min before being washed with PBS. After being washed with PBS, the sectioned tissues were incubated with primary antibodies. Excess antibodies were washed away with PBS. Secondary antibodies (progesterone (PR) Cat. No. 33-189; estrogen (ER) Cat. No. 06-935; Ki-67 cat. No. IHC-00375; human epidermal growth factor receptor 2 (HER-2) Cat. No. MA5-13070; pan-cytokeratin Cat. No. C2931; smooth muscle actin (SMA) Cat. No. 23081-1-AP; and desmin Cat. No. D1033) were introduced for 15 min. The addition of horseradish peroxidase (HRP) was done for 15 min. The working solution (a drop of 3,3′-diaminobenzidine (DAB) chromogen and 1 ml of the DAB substrate) was applied to the sectioned tissues after washing off the HRP with PBS for 5 min.

### Tissue Preparation and Homogenization

The Wistar rats were sacrificed by cervical dislocation. The blood was obtained through the abdominal vein and centrifuged at 2,500 rpm for 10 min. A portion of the harvested tumors was stored in PBS at −4°C and homogenized. The homogenates were centrifuged at 4,000 rpm for 10 min. The supernatants were used for the biochemical assays. The tumors were also excised for mRNA expression, while some were stored in 10% buffered formalin.

### Biochemical Analysis

#### Creatinine and Urea Concentration Determination

The Randox CR510 kit was used for creatinine and urea concentration assessment in the serum of groups 1–6 Wistar rats.

### Reverse Transcription–Polymerase Chain Reaction

RNA isolation from the mammary tumors, reverse transcription, and quantitative PCR were carried out as earlier reported by Akinloye et al. (7). RNAs were isolated from the tumors with TRIzol reagent. The RNAs were dissolved with RNA-free DNase and purified with the RNeasy kit (Qiagen, Germany). Reverse transcriptase, random hexanucleotides, and 40 μg of total RNA were incubated at 37°C for 60 min to synthesize cDNA. The primers were designed with the Snap gene software. Glyceraldehyde 3-phosphate dehydrogenase (GAPDH) was used as the control gene. The thermocycler was used to amplify the mRNAs at 50 cycles for 2 h 20 min. The PCR products were run on 1.0% agarose gels. Ethidium bromide (EtBr) staining was used for visualization. The primers are as follows:

**Table:** 

Target genes	Forward 5′–3′	Reverse 5′–3′
**GAPDH**	AAGGGCTCATGACCACAGTC	GGATGCAGGGATGATGTTCT
**p27**	ACTCTGAGGACCGGCATTTG	CATTCGGGGAACCGTCTGAA
**p53**	CCCCTGAAGACTGGATAACTGT	TCTCCTGACTCAGAGGGAGC
**HER2**	ATCATCATGGAGCTGGCGGC	TGTCCAGGTGGGTCTCAGGA

### Histoarchitectural Analysis of Mammary Tumor

The tumors were fixed in formal saline (10%). The fixative was removed by running water through the tumors. The tumors were dehydrated and washed in methyl-benzoate and later soaked in paraffin. They were cut into 3- to 5-μm thickness and stained with hematoxylin and eosin (H&E).

### Statistical Analysis

The data were expressed as the means ± standard error of the mean (SEM). One-way analysis of variance (ANOVA) in GraphPad Prism 7 was used. Statistical significance equals *p* < 0.05.

## Results

### LC-ESI-MS Validated the Carotenoids

**Figure 1:**
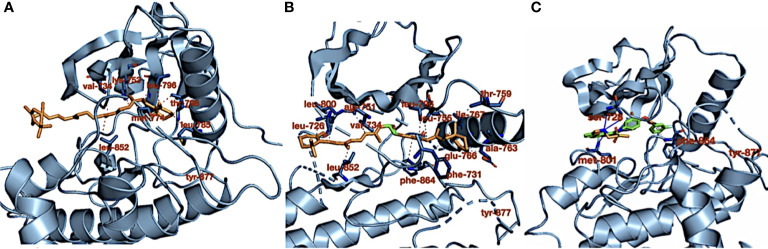
**(A)** Crystal structure of HER2 kinase domain in combination with **(A)** 7,7′,8,8′-tetrahydro-β,β-carotene (orange), **(B)** 7,7′,8,8′-tetrahydro-β,β-carotene (orange), and **(C)** lapatinib (green). The dotted red and gray lines (in **B**) represent hydrophobic interactions, the blue lines represent hydrogen bond interactions, and the dotted gray line (in **C**) represents pi stacking.

**Table 1** shows the carotenoid fragments obtained from the LC-ESI-MS. The compounds are astaxanthin, beta-carotene-15,15′-epoxide (BTC), and 7,7′,8,8′-tetrahydro-β,β-carotene (TTC) beta-carotene-15,15’-epoxide (BTC) (**Table 1**) (8). **Figure 1** showed the molecular interactions of BTC, TTC, and lapatinib within the ATP kinase domain of HER2.

**Table 1:** Fragments of positive ionization of the carotenoid-enriched fractions.

Identification	Elemental composition	Molecular ion [M^+^H^+^]	MS/MS fragmentation (positive ion mode) *m*/*z*	References
a. Astaxanthin	C_40_H_52_O_4_	600.5559	311, 283, 166, 88	(23)
b. beta-carotene-15,15′-epoxide	C_40_H_56_O	552.4996	429, 307, 81	(23, 24)
c. 7,7′,8,8′-Tetrahydro-β,β-carotene	C_40_H_60_	540.5347	311, 165, 102	(23)

### Carotenoid-Enriched Fractions Demonstrate Inhibition of HER2 ATP Kinase Domain

**Figure 2:**
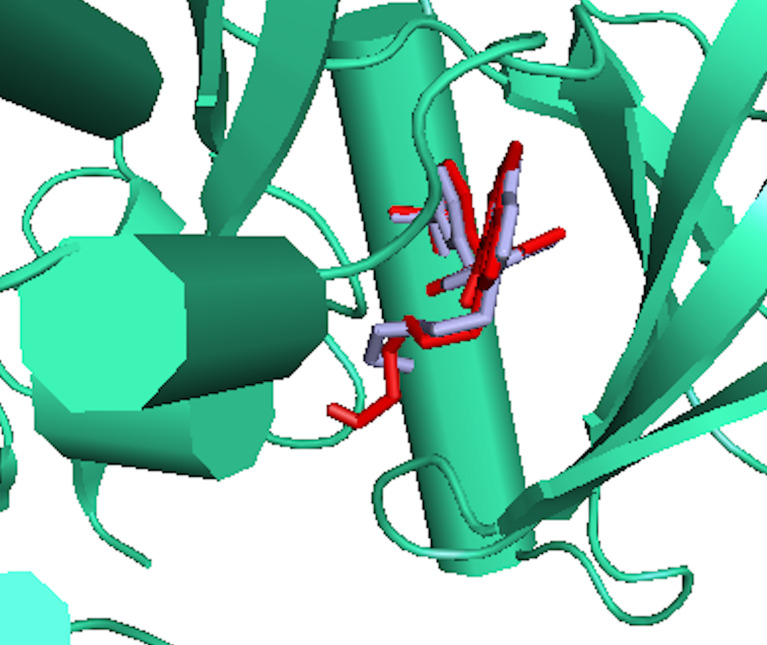
The binding poses of the re-docked (red) and the co-crystallized, 2-{2-[4-({5-chloro-6-[3-(trifluoromethyl) phenoxy] pyridin-3-yl} amino)-5H-pyrrolo[3,2-d] pyrimidin-5-yl] ethoxy} ethanol (light blue) within the ATP kinase domain of 3PP0.

**Figure 3:**
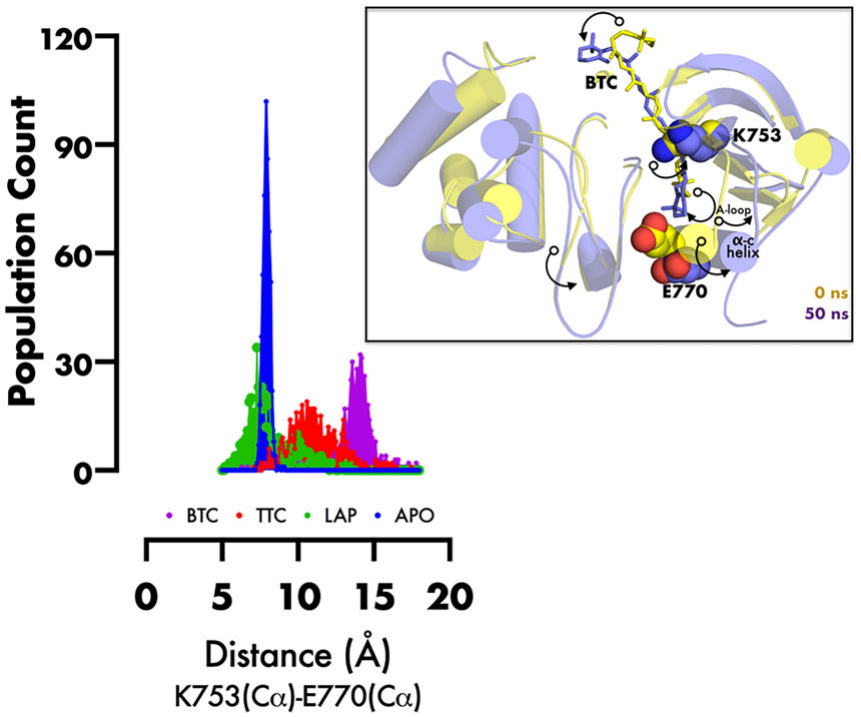
Frequency distribution plot of the inter-atomic distance between K753 and E770 (superimposed initial and final structures of BTC-bound HER2, showing the movement of specific regions of protein (black curved arrows) during inactivation). PyMol was used for 3D representation.

RMSD of the re-docked was 0.071 Å (**Figure 2**) (25). **Table 2** showed the docking scores of TTC , BTC, and LAP within the ATP kinase domain of 3PP0. BTC has a docking score of -11.5 kcal/mol, TTC has a docking score of -8.5 kcal/mol, astaxanthin has a docking score of -3.0 kcal/mol, while lapatinib, the standard drug has a docking score of -10.6 kcal/mol. With the use of frames obtained for every 50 ps, over 50 ns time, MM/GBSA method was used to estimate the binding energies of TTC, BTC in comparison with lapatinib (LAP) (**Table 3**). The result shows that BTC has the lowest mean binding energy (dG binding = −52.70 kcal/mol), followed by TTC (dG binding = −37.72 kcal/mol) and LAP (dG binding = −26.82 kcal/mol). Furthermore, the frequency distribution curve shows that the APO state HER2 sampled several conformations with fully formed salt bridge between K753 and E770 (Ca–Ca distance < 6 Å). The standard drug LAP also demonstrated its ability to break the salt bridge in almost 30% of the conformations (green curve), while the two compounds from *S. mombin* effectively ruptured K753 and E770 salt bridge (Ca–Ca distance > 7 Å) during the simulation (**Figure 3**). A superimposition of the initial starting structure (0 ns) and the last structure generated (50 ns) of HER2 in BTC-bound state provides a structural insight into the possible atomistic basis for the ruptured salt bridge. Here, the trimethyl-substituted cyclohexene-1-yl rings on both ends of BTC flipped into the core of the protein in the 50-ns structure (flipped black arrows), indicating stronger ligand binding. On the other hand, the a-C helix and the A-loop moved away from the active site region, thus allowing E770 to move its carboxylate moiety away from the e-amino group of K753, rupturing the salt bridge (**Figure 3**).

**Table 2:** Docking score of the carotenoid-enriched fractions against HER2.

Carotenoid-enriched fractions/standard drug	Docking score with HER2 kinase domain (kcal/mol)
Astaxanthin	−3.0
7,7′,8,8′-Tetrahydro-β,β-carotene	−8.5
beta-carotene-15,15′-epoxide	−11.5
Lapatinib	−10.6

**Table 3:** MM/GBSA-estimated free energies of binding.

Ligand	dE (internal) (kcal/mol)	dE (electrostatic) + dG (sol) (kcal/mol)	dE (VDW) + dG (sol) (kcal/mol)	dG (binding) (kcal/mol)
LAP	2.038	13.37	−42.23	−26.82
BTC	−2.32e^−05^	20.08	−72.78	−52.70
TTC	1.080e^−04^	14.81	−52.52	−37.72

MM/GBSA, molecular mechanics/generalized Born surface area; LAP, lapatinib; BTC, beta-carotene-15,15′-epoxide; TTC, 7,7′,8,8′-tetrahydro-β,β-carotene; VDW, Van der Waals.

### The Downregulation of HER2 mRNA Validated the *In Silico* Result

**Figure 4:**
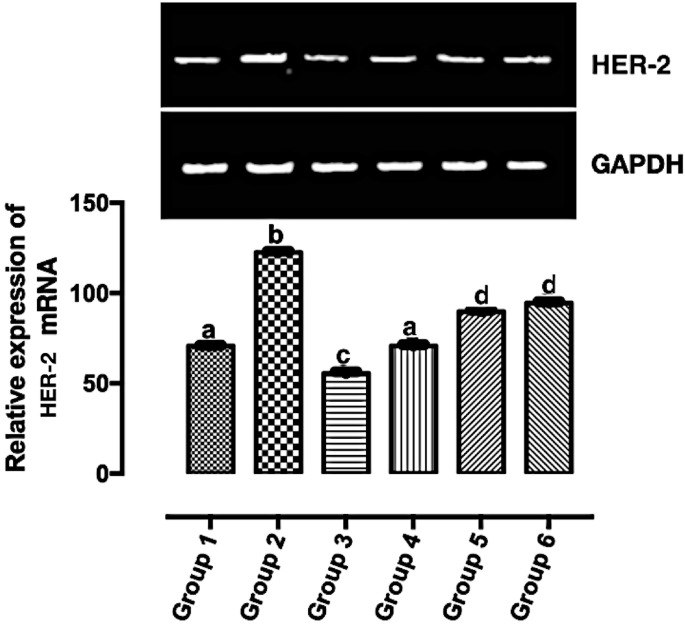
Relative expression of HER2 mRNA in the mammary tumor of Wistar rats treated with the carotenoid-enriched fractions. Different letters on the bars show significant difference. Significance is at *p* < 0.05.

HER2 is overexpressed in a good number of breast cancer. Its overexpression corresponds to a poor prognosis and increased mortality (26). HER2 mRNA expression was significantly downregulated (*p* < 0.05) in all the carotenoid-rich fraction-treated groups when compared with the untreated group (group 2) (**Figure 4**). There was no significant difference in the expression of HER-2 mRNA in groups 5 and 6. The expression of HER-2 mRNA was significantly upregulated in in groups 5 and 6 when compared with groups 3 and 4. The expression of HER-2 mRNA was significantly downregulated in group 3 when compared with groups 1, 2, 4, 5, and 6.

### The Upregulation of p53 and p27 mRNAs Validate the *In Silico* Results

**Figure 5:**
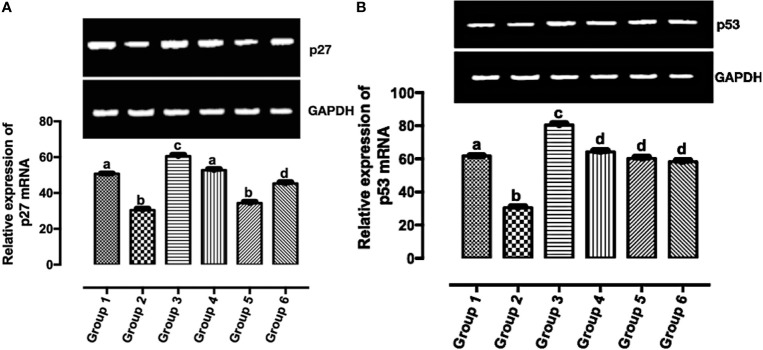
Relative expression of **(A)** p53 and **(B)** p27 mRNAs in the mammary tumor of Wistar rats treated with the carotenoid-enriched fractions. Different letters on the bars show significant difference. Significance is at *p* < 0.05.

According to Casalini et al. (27), a correlation exists between p53 and p27 expression with HER2 signaling. In **Figure 5A**, the expression of p27 mRNA was significantly upregulated (*p* < 0.05) in the treatment groups except in group 5 when compared with group 2. The significant expression of p27 mRNA was greater in group 3 when compared with other groups (groups 1, 2, 4, 5, and 6). There was no significant difference in the expression of p27 mRNA in group 1 and group 4. Also, there was no significant difference in the expression of p27 mRNA in group 2 and group 5. In **Figure 5B**, the significant expression of p53 mRNA was greater in group 3 when compared with other groups (groups 1, 2, 4, 5, and 6). There was no significant difference in the expression of p53 mRNA in groups 4–6. The expression of p53 mRNA was significantly downregulated (*p* < 0.05) in group 2 when compared with the other groups.

### Immunohistochemistry Validate Model of HER2^+^ Breast Cancer Subtypes

**Figure 6:**
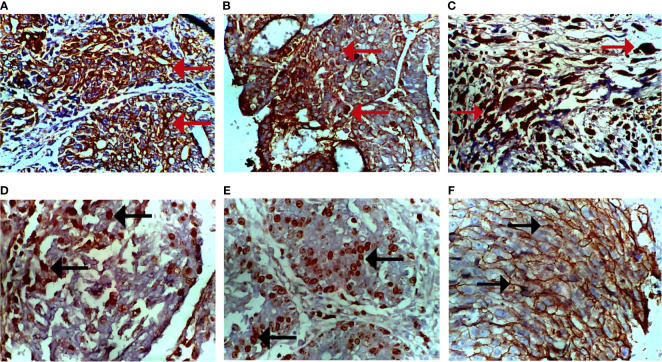
Photomicrograph of breast sections. **(A)** Moderate expression of pan-cytokeratin on malignant cells (cytoplasmic stain; marked by red arrows). **(B)** Moderate expression of CK5 on epithelioid cells of the breast (cytoplasmic stain; marked by red arrows). **(C)** Diffused and high expression of smooth muscle actin (SMA) by malignant cells (nuclear stain; marked by red arrows). **(D)** Diffused and moderate expression of desmin by malignant cells (nuclear stain; marked by black arrows). **(E)** Dispersed and mild expression of Ki67 by malignant cells (nuclear stain; marked by black arrows). **(F)** Diffused and moderate expression of HER-2 of malignant cells (cytoplasmic stain; marked by black arrows). Immunohistochemical stained tissue sections. Positivity is indicated by brown-stained cells. Magnification, ×400.

Immunohistochemical analysis of the tumors using PR, ER, Ki-67, HER-2, pan-cytokeratin, SMA, and desmin antibodies. **Figure 6A** shows a moderate expression of pan-cytokeratin on malignant cells of the breast (marked by red arrows), **Figure 6B** shows high expression of cytokeratin 5 (CK5) on epithelial cells overlying mammary glands (marked by black arrows), **Figure 6C** shows diffused and high expression of SMA by connective tissue cells (sarcoma; indicated by brown-stained cells), **Figure 6D** also shows diffused and high expression of desmin by connective tissue cells (sarcoma; indicated by brown-stained cells), **Figure 6E** shows dispersed and mild expression of Ki67 by malignant cells of the breast (marked by black arrows), and **Figure 6F** shows diffused and moderate expression of HER-2 by malignant cells of the breast.

### Carotenoid-Enriched Fractions Reduced Tumor Volume

**Figure 7:**
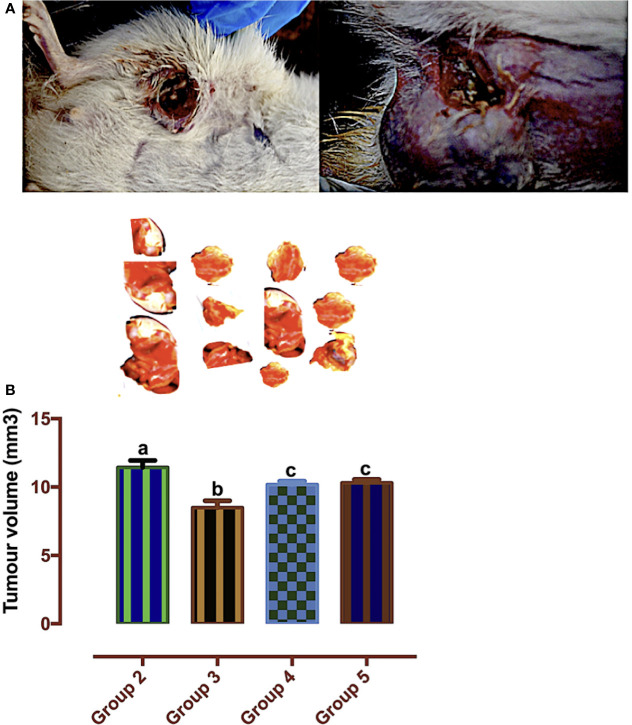
**(A)** Mammary tumors. **(B)** Tumor volumes of Wistar rats treated with the carotenoid-enriched fractions. Different letters on the bars show significant difference. Significance is at *p* < 0.05.

According to Mozley et al. (28), changes in tumor volume are biomarkers for response to treatment. In **Figure 7**, the tumor volume was significantly reduced in the carotenoid-rich fraction-treated groups when compared with group 2. There was no significant difference in the tumor volume of group 4 and group 5. The tumor volume of group 3 was significantly reduced when compared with that of the other groups (2, 4, and 5).

### Carotenoid-Enriched Fractions Attenuated Serum Creatinine and Urea Concentrations

**Figure 8:**
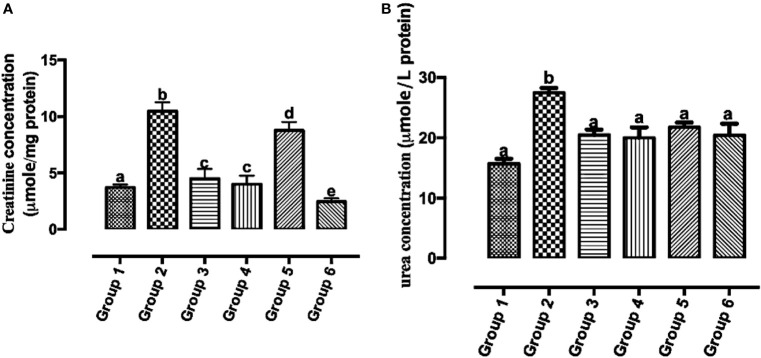
Creatinine **(A)** and urea **(B)** concentrations in the serum of Wistar rats treated with the carotenoid-enriched fractions. Different letters on the bars show significant difference. Significance is at *p* < 0.05.

Reports of the existence of increased serum creatinine levels and poor prognosis in individuals with breast cancer have been established (29). The urea cycle increases nitrogen consumption in pyrimidine biosynthesis in cancer, and elevated urea concentration is connected to poor prognosis in neoplasm (30). In **Figure 8A**, there was a significant reduction in all the carotenoid-rich fraction-treated groups when compared with the untreated group (group 2). There was no significant difference in serum creatinine levels of group 3 and group 4. There was a significant reduction in serum creatinine levels in group 6 when compared with other groups (1, 2, 3, 4, and 5). **Figure 8B** shows that the urea concentration was significantly increased in group 2 when compared with groups 1, 3, 4, 5, and 6. Urea concentration was significantly attenuated in the treatment groups when compared with group 2. There was no significant difference in the urea concentration of groups 3–6.

### DMBA Initiated Subtypes of Breast Cancer

**Figure 9:**
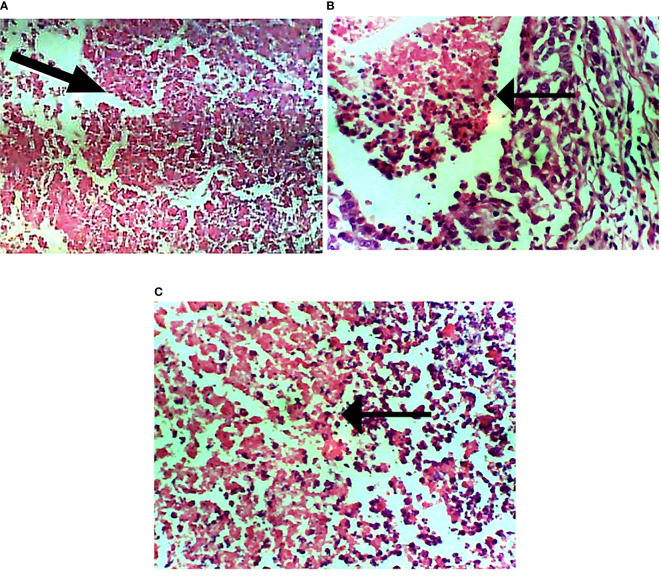
Photomicrograph of breast sections. **(A)** Extensive necrosis of malignant cells (marked by black arrow). **(B)** Mild focal necrosis of malignant cells. **(C)** Moderate necrosis of malignant cells. Hematoxylin and eosin-stained tissue sections. Magnification, 400.

The subtypes of breast cancer dictate the forms of treatments. H&E staining revealed the HER2^+^ breast cancer is both comedo and ductal carcinoma. The treatment induced apoptosis (**Figure 9**).

## Discussion

The rate of mortality from breast cancer continues to be on the rise, especially in developing countries. In this study, we showed that carotenoid-enriched fractions from *S. mombin* demonstrate anti-HER2 positive breast carcinoma potentials *via* HER2 ATP kinase domain inhibition.

As shown in our previous study, the phytoconstituents of the carotenoid-enriched fractions are astaxanthin, BTC, and 7,7′,8,8′-tetrahydro-β-β-carotene (8). They were docked into the ATP kinase domain of HER2. BTC has the highest docking score and binding energy of −11.5 and −52.70 kcal/mol respectively. 7′,8,8′-Tetrahydro-β,β-carotene (TTC) has a docking score of −8.5 kcal/mol and binding energy of −37.72 kcal/mol. Astaxanthin, on the other hand, showed little inhibition of the HER2 kinase domain, with a docking score of −3.0 kcal/mol. The standard drug, lapatinib (LAP), has a docking score of −10.6 kcal/mol and binding energy of −26.82 kcal/mol. It is worthy of note that both BTC and TTC showed better inhibition of the kinase domain than LAP (as revealed by the binding energies). BTC spans through the whole length of the ATP binding domain, forming extensive 13 hydrophobic interactions (leu-726, leu-800, ala-751, val-734, leu-796, leu-755, ile-767, thr-759, ala-763, glu-766, phe-731, phe-864, and leu-852) within the ATP binding domain. This might be responsible for its better inhibition of the HER2 kinase domain when compared with TTC with seven hydrophobic interactions (val-734, lys-753, leu-796, thr-798, met-774, leu-785, and leu-852) within the ATP binding domain. It is noteworthy that lapatinib, though with fewer interactions within the ATP domain, has a very high inhibitory power probably due to the formation of three hydrogen bonds (ser-728, ser-728, and met-801) and one pi stacking bond (phe-864). Hydrogen bond interactions seem to favor the inhibition of the HER2 kinase domain. Re-docking of the co-crystallized into the ATP binding domain of 3PP0 gave an RMSD value of 0.071 Å (23). The re-docked pose reproduced the experimental pose; this shows that the docking scores obtained in this study are accurate.

The kinase domain of HER2 exists in two structurally distinct states: active and inactive states (29). The active state is characterized by the presence of a salt bridge formed by two evolutionarily conserved amino acid residues K753 and E770. This canonical feature is absent in inactive state conformation (21), especially HER2 in the inhibitor-bound state, such as that used in the docking studies (21). This, therefore, begs the question, how do the inhibitors proposed from *S. mombin* affect the salt bridge? To answer this question, the HER2 model built as an active state (13) conformation was subjected to atomistic simulation, and the Ca distance between the K753/E770 pairs was monitored in apo- and inhibitor-bound states. The two compounds from *S. mombin* not only ruptured the salt bridge but moved the α-helix C away from the nucleotide phosphate-binding loop. A key implication of α-helix C movement from its active state position is the disruption of the allosteric activation *via* dimerization. In dimerization-induced activation of HER2, the α-helix C in a monomer-A must make hydrophobic interactions with residues in the C-lobe of monomer-B; such interactions are therefore potentially disrupted by two compounds from *S. mombin*, thus making them potent inhibitors of the HER2 kinase domain (21, 31, 32).

**Figure 10:**
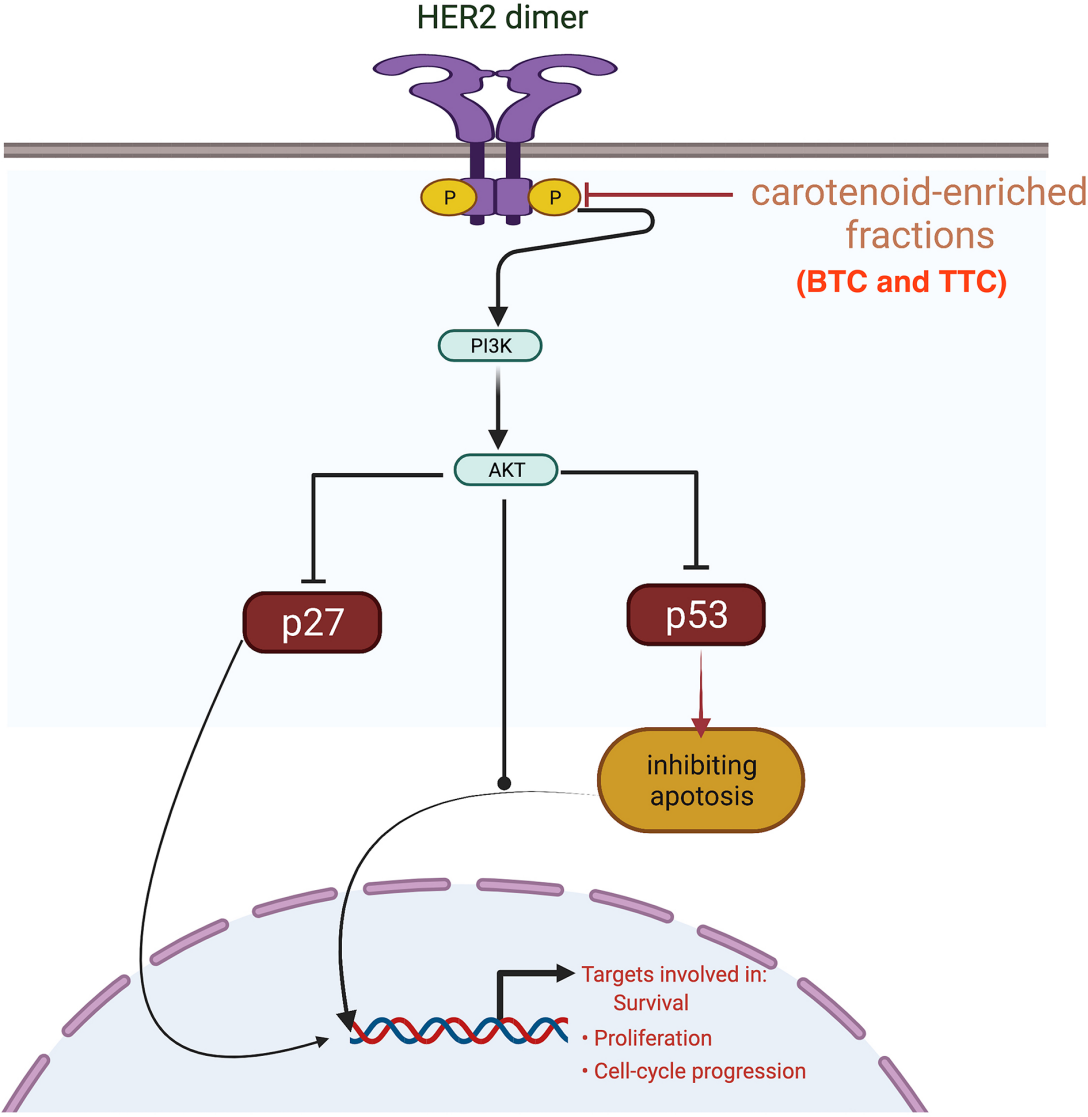
Mechanism of inhibition of the kinase domain by the carotenoid-enriched fractions in HER2 signaling pathway.

HER2 mRNA expression was downregulated by the carotenoid-enriched fractions, depicting inhibition of HER2 ATP kinase domain as revealed in the *in silico* study. This observation also corroborated the reported anticancer potentials of carotenoids (33). According to Casalini et al. (27), there is a correlation between p53 and p27 expression with HER2 signaling. One of the mechanisms of HER2 signaling is through the phosphatidylinositol 3-kinase (PI3K)/protein kinase B (Akt) signaling pathway involving p53 and p27 (34). The upregulation of p53 and p27 mRNAs by the carotenoid-enriched fractions corroborated the report of McDermott et al. (35) that there is an upregulation of p53 and p27 mRNAs when HER2^+^ breast cancer is treated with tyrosine kinase inhibitor (TKI). Hence, we proposed that the inhibition of the kinase domain by the carotenoid-enriched fractions is through the PI3K/AKT downstream signaling (**Figure 10**).

Immunohistochemical staining of tumors was positive for HER-2 antibody but negative for CK5 and ER. This validated the HER2^+^ model of breast cancer subtypes in this study. It is noteworthy that the present study fails to support earlier reports that have hitherto hypothesized DMBA as a model of ER-dependent breast cancers (36). The immunohistochemical stains were positive for pan-cytokeratin and CK5, confirming the presence of epithelial cells. Hence, the neoplastic growth is epithelium-originated; that is, it is a carcinoma. The tumors were positive for both SMA and desmin sarcoma. This revealed that DMBA induces sarcoma as well as comedo carcinoma. The expression of Ki67 was mild as a result of treatment with carotenoid-enriched fractions demonstrating its anti-proliferating tendency (37).

Determination of the tumors volume is proven to have prognostic benefits (38). Treatment with the carotenoid-enriched fractions significantly reduces the tumors’ volume when compared with that in group 2. This is likely due to the anti-HER2^+^ potentials of the carotenoid-enriched fractions. This observation is in tandem with the study of Chew et al. (39) that carotenoids attenuated tumor volume.

Reports of the existence of increased serum creatinine levels and poor prognosis in individuals with breast cancer have been established (29). The urea cycle increases nitrogen consumption in pyrimidine biosynthesis in cancer, and elevated urea concentration is connected to poor prognosis in neoplasm (30). The reduction in the concentration of serum creatinine and urea concentration by the treatments further gives credence to the anticancer tendencies of the carotenoid-enriched fractions.

The formation of extensive necrosis due to the treatment is in tandem with the report of Amaravadi and Thompson (40) that necrosis can be a result of apoptosis. The HER2-positive model of breast cancer is of comedo and invasive ductal subtypes. It is worthy of note that the treatment (carotenoid-enrich fractions) does not have any negative effects on non-tumorigenic Wistar rats as revealed in group 6 (**Figures 4**, **5**, and **8**).

## Conclusion

Carotenoid-enriched fractions from *S. mombin* inhibit the ATP kinase domain of HER2 in *silico* studies and validated *via in vivo* studies by downregulating HER2 mRNA expression and upregulating p53 and p27 mRNA expression in the HER2^+^ model of breast cancer. The HER2^+^ model of breast cancer is of comedo and invasive ductal subtypes. The carotenoid-enriched fractions also attenuate serum creatinine and urea concentrations and reduce the tumor volume of the HER2^+^ model of breast carcinoma. The carotenoid-enriched fractions demonstrate anti-HER2 positive breast carcinoma potentials *via* HER2 ATP kinase domain inhibition.

## Data Availability Statement

The original contributions presented in the study are included in the article/supplementary material, further inquiries can be directed to the corresponding author.

## Ethics Statement

The animal study was reviewed and approved by Federal University of Agriculture Abeokuta Animal Ethics Board.

## Author Contributions

DSM proposed, took part in all aspects of the study and wrote the manuscript. OOA designed, supervised the study and edited the manuscript. OIO wrote part of the manuscript, carried out the molecular dynamic simulation studies, and analyzed the data. JOO carried out the immunohistochemical and histological analysis. MAP carried out the *in-silico* screening and participated in the molecular gene expression profiling. AJA participated in the molecular gene expression profiling. All authors contributed to the article and approved the submitted version.

## Funding

This research is supported in part by a grant from Afe Babalola University, Ado-Ekiti (ABUAD), Nigeria, and in part by a grant from Tertiary Education Trust Fund (TETFUND), Nigeria.

## Conflict of Interest

The authors declare that the research was conducted in the absence of any commercial or financial relationships that could be construed as a potential conflict of interest.

## Publisher’s Note

All claims expressed in this article are solely those of the authors and do not necessarily represent those of their affiliated organizations, or those of the publisher, the editors and the reviewers. Any product that may be evaluated in this article, or claim that may be made by its manufacturer, is not guaranteed or endorsed by the publisher.
